# Optimization of Phenolic‐ and Saponin‐Enriched Extraction From *Pandanus tectorius* Fruit Using Box–Behnken Design and Evaluation of Their Bioactivities

**DOI:** 10.1155/jamc/5539843

**Published:** 2025-12-09

**Authors:** Do Hoang Giang, Nguyen Hai Dang, Tran Thi Thu Phuong, Le Thanh Huong, Nguyen Thu Uyen, Nguyen Thi Luyen, Nguyen Thi Thu Thuy, Hoang Le Tuan Anh, Nguyen Ngoc Tung, Nguyen Tien Dat

**Affiliations:** ^1^ University of Science and Technology of Hanoi, Vietnam Academy of Science and Technology, Hanoi, 10000, Vietnam, usth.edu.vn; ^2^ Center for High Technology Research and Development, Vietnam Academy of Science and Technology, Hanoi, 10000, Vietnam, vast.ac.vn; ^3^ Department of Pharmacy, Joint Vietnam-Russia Tropical Science and Technology Research Center, Hanoi, 10000, Vietnam

**Keywords:** anti-inflammation, antioxidant, Box–Behnken, multiresponse optimization, *Pandanus tectorius*, response surface method

## Abstract

*Pandanus tectorius* fruits are a promising but underutilized source of bioactive constituents. We optimized extraction conditions for phenolic‐ and saponin‐enriched fractions using Box–Behnken/response surface methodology across ethanol concentration, temperature, solvent‐to‐material ratio, and time and then evaluated antioxidant and anti‐inflammatory activities. Total phenolic content (TPC; Folin–Ciocalteu, 760 nm) and total saponin content (TSC; vanillin–sulfuric acid, 560 nm) served as responses for model fitting (*R* ^2^ > 0.96), validation, and multiresponse optimization that yielded seven distinct optimums targeting different extract profiles. Phenolic‐rich extracts showed potent DPPH and hydroxyl radical scavenging, whereas saponin‐rich extracts more strongly inhibited LPS‐induced nitric oxide in RAW 264.7 cells; excessive saponin enrichment, however, coincided with cytotoxicity. These results demonstrate that tuned extraction can deliver purpose‐built extracts for antioxidant or anti‐inflammatory applications, supporting the valorization of *P. tectorius* as a natural source for functional and nutraceutical ingredients.

## 1. Introduction

The genus *Pandanus* (family Pandanaceae) comprises approximately 700–750 species predominantly distributed across tropical and subtropical regions of the Paleotropics, extending from West Africa to the Pacific Islands [1, 2]. Members of this genus are morphologically distinctive, characterized by spiral phyllotaxy, long, narrow leaves, and prominent aerial prop roots that aid in anchorage and adaptation to coastal or sandy habitats [1]. Ecologically, *Pandanus* species contribute significantly to shoreline stabilization and serve as key components in tropical forest ecosystems. Culturally, they hold economic and ethnobotanical value, with their leaves widely used in traditional crafts (e.g., mats, baskets, and roofing materials) and, in some regions, as aromatic ingredients in culinary practices [1, 2].

*Pandanus tectorius* Parkinson ex Du Roi, a member of the Pandanaceae family, is widely distributed throughout tropical and subtropical coastal regions of Asia and the Pacific. It has long been used in traditional medicine for the treatment of ailments such as hypertension, diabetes, urinary tract infections, and inflammation. Recent phytochemical investigations have revealed that this species is a rich source of structurally diverse secondary metabolites with notable biological activities. Key compound classes identified include phenolic acids and aldehydes (e.g., p‐coumaric acid, ferulic acid, vanillin, and syringaldehyde), flavonoids (e.g., vitexin, tricin, chrysin, and sakuranetin), and various lignans such as pinoresinol, syringaresinol, medioresinol, eudesmin, and sesamin, which have been isolated from fruits, roots, and leaves [3–8]. Coumarins, including bergapten and several prenylated derivatives, have also been detected, along with one benzofuran compound and multiple volatile constituents such as geranyl acetate and ethyl cinnamate identified by GC‐MS analysis [4, 7, 8]. Furthermore, several miscellaneous compounds such as 5‐hydroxymethylfurfural, methylsuccinic acid, long‐chain fatty alcohol esters, and sugar derivatives were also reported [6, 9–12].

A growing body of evidence has demonstrated that *P. tectorius* possesses a wide range of biological activities, including antioxidant, cytotoxic, antimicrobial, and antidiabetic effects. Among the phytoconstituents, phenolic compounds stand out as the most studied and bioactive group. Several in vitro assays, such as DPPH, ABTS, and hydroxyl radical scavenging tests, have confirmed the strong antioxidant capacity of both crude extracts and phenolic‐enriched fractions, especially those obtained using ethyl acetate or methanol [7–10, 13]. These effects are attributed to abundant phenolic aldehydes and acids, flavonoids, and lignans found in fruits and leaves. For instance, caffeoylquinic acid derivatives from *P. tectorius* have shown lipid‐lowering effects in hyperlipidemic hamsters via modulation of PPARα and AMPK signaling pathways [14]. Moreover, phenolic constituents were reported to protect Schwann cells from oxidative stress through activation of the Nrf2/Keap1 antioxidant response pathway [15]. Antidiabetic potential has also been widely explored through α‐glucosidase inhibition assays. Multiple phenolic compounds—including aromatic aldehydes, coumarins, and flavonoids—exhibited strong inhibitory activity and promising hypoglycemic effects [6, 9, 10]. In addition, extracts of *P. tectorius* have demonstrated selective cytotoxicity against various human cancer cell lines such as A549, MCF‐7, and HeLa, with dose‐dependent inhibition of cell viability [8, 16, 17]. Furthermore, antimicrobial activity has been observed in both polar and semipolar extracts, effective against a range of gram‐positive and gram‐negative bacteria [7]. Recent developments also explored the use of *P. tectorius* fruit extract in nanoparticle formulations for potential therapeutic applications in metabolic disorders [16].

Despite extensive research on phenolics, the saponins of *P. tectorius* remain largely underexplored. Saponins in *Pandanus* species are expected to comprise triterpenoid and steroidal frameworks, consistent with related genera, and have been associated with membrane‐active, hemolytic, and immunomodulatory properties relevant to inflammation. Preliminary studies in *P. tectorius* and allied species have suggested that saponin‐containing fractions contribute to anti‐inflammatory or cytoprotective effects, yet systematic enrichment, quantification, and modeling remain limited [15]. This knowledge gap highlights the need for targeted extraction and comparative evaluation of both phenolic and saponin constituents to better understand their relative contributions to bioactivity.

Because extraction efficiency is determined by complex interactions among solvent polarity, temperature‐driven mass transfer and degradation kinetics, solvent‐to‐material ratio, and extraction time, the traditional one‐factor‐at‐a‐time approach is inadequate for identifying true optima. Response surface methodology (RSM), particularly the Box–Behnken design (BBD), enables quantitative assessment of main, interaction, and curvature effects while reducing experimental runs and improving predictive accuracy. Therefore, the present study employed RSM/BBD to optimize the extraction of phenolic‐ and saponin‐enriched fractions from *P. tectorius* fruits, aiming to maximize both yield and bioactivity through multiresponse optimization.

## 2. Materials and Methods

### 2.1. Plant Materials

Fruits of *P. tectorius* were collected at Thanh Oai Province, Vietnam, in August 2021 and identified by Dr. Bui Van Thanh, Institute of Biology, Vietnam Academy of Sciences and Technology (VAST). A voucher specimen (NCCG 210213) was deposited at the Center for High Technology Research and Development, VAST. The collected sample was cleaned, dried at 60°C in the oven to under 10% moisture, milled, sieved to < 1 mm to improve batch homogeneity and mass transfer reproducibility prior to extraction, and preserved at −20°C for further experiments.

### 2.2. General

The solvents, such as ethanol (EtOH), water, DMSO, and necessary inorganic chemicals, were purchased from Daihan Scientific, Korea; meanwhile, other chemicals were supplied by Merck, Germany. The extraction was processed in a shaking water bath with the support of a stirrer (Daihan Scientific, Korea).

### 2.3. Determination of Total Phenolic Content (TPC)

The TPCs of the samples were determined using the Folin–Ciocalteu assay [18]. The characteristic blue chromophore also provided qualitative confirmation of phenolics in the samples. Standard solutions of gallic acid at various concentrations were prepared for calibration. Sample extracts were dissolved in methanol at defined concentrations. A volume of 100 μL from each sample or standard solution was combined with 900 μL of 10% Folin–Ciocalteu reagent and 1000 μL of 6% sodium carbonate (Na_2_CO_3_). The resulting mixture was incubated at 40°C for 15 min. Absorbance was measured at 760 nm using a UV–visible spectrophotometer. The TPC was quantified based on the gallic acid standard curve and expressed as milligrams of gallic acid equivalents per gram of sample (mg GAE/g).

### 2.4. Determination of Total Saponin Content (TSC)

The TSC of the samples was assessed using the vanillin–sulfuric acid method [19]. The characteristic reddish (vanillin–H_2_SO_4_) chromophore also indicated qualitative confirmation of saponins in the samples. For this procedure, 100 μL of each extract was mixed with 100 μL of 8% (w/v) vanillin in EtOH and 2800 μL of 80% (v/v) sulfuric acid. The mixture was incubated at 70°C for 15 min. Solutions of the reference compound (aescin) and reagent blanks (with solvent) were also prepared. After incubation, the mixtures were allowed to cool at room temperature for 5 min, and absorbance was recorded at 560 nm against the blank.

### 2.5. Preliminary Single‐Factor Experiments

Preliminary single‐factor experiments were conducted to establish the appropriate ranges for key extraction parameters, including temperature, EtOH concentration, solvent‐to‐material ratio, and extraction time. First, the influence of EtOH concentration on TPC and TSC was examined by performing extractions with EtOH concentrations ranging from 0% to 90%, at 60°C for 120 min, maintaining a constant solvent‐to‐material ratio of 20 mL/g. Subsequently, the effect of extraction temperature on the TPC and TSC was investigated by extracting the plant material in 60% EtOH at temperatures ranging from 30°C to 100°C for 120 min, using a solvent‐to‐material ratio of 20 mL/g. Next, the effect of varying the solvent‐to‐material ratio was assessed by extracting the material in 60% EtOH at 60°C for 120 min, using ratios ranging from 5 to 50 mL/g. Finally, the impact of extraction time was evaluated by extracting the plant material in 60% EtOH at 60°C with a fixed solvent‐to‐material ratio of 20 mL/g for durations ranging from 60 to 360 min.

### 2.6. Response Surface Method

To optimize the extraction conditions for phenolic enrichment from *P. tectorius* fruits, RSM based on the BBD was employed. The experimental design was carried out using Design‐Expert software version 12.0 (Stat‐Ease, Inc., Minneapolis, USA). Four independent variables were selected: extraction temperature (°C, A), EtOH concentration (%, B), solvent‐to‐material ratio (mL/g, C), and extraction time (minutes, D). The TPC and TSC were designated as the response variables.

Based on the results of preliminary single‐factor experiments, the solvent system included EtOH concentrations of 0% (distilled water), 45%, and 90%. The temperature range tested was 30°C–80°C, the solvent‐to‐material ratios ranged from 10 to 50 mL/g, and the extraction times varied between 60 and 240 min. All experimental runs were performed in triplicate, and the average value of TPC was used for subsequent statistical analysis. The coded levels of the independent variables used in the experimental design are presented in Table 1.

**Table 1:** Coded and actual levels of independent variables used in the Box–Behnken design.

Variables	Unit	Code levels		
		−1	0	1
Temperature (A)	°C	30	55	80
Ethanol concentration (B)	%	0	45	90
Volume‐to‐weight ratio (C)	mL/g	10	30	50
Extraction time (D)	min	60	150	240

### 2.7. Antioxidant Assay

The antioxidant potential of the phenolic‐enriched extracts was assessed using DPPH and hydroxyl radical scavenging assays, following established protocols [20, 21].

For the DPPH assay, 100 μL of each sample was mixed with 1900 μL of DPPH solution in methanol and incubated in the dark at 37°C for 20 min. Absorbance was measured at 517 nm, with ascorbic acid serving as the reference standard.

In the hydroxyl radical scavenging assay, a 200 μL aliquot of each test sample was added to a mixture containing 400 μL of 50 mM phosphate buffer (pH 7.8), 400 μL of 2.8 mM deoxyribose, and 400 μL of 500 μM ferrous ammonium sulfate [Fe(NH_4_)_2_(SO_4_)_2_]. The mixture was incubated at 37°C for 1 h. The reaction was terminated by the addition of 1000 μL of 10% (w/v) trichloroacetic acid and 1000 μL of 1% (w/v) thiobarbituric acid. The resulting solution was then heated in a boiling water bath for 15 min, and absorbance was measured at 532 nm. Catechin was used as the positive control for this assay.

### 2.8. Nitric Oxide (NO) Production Inhibition Assay

The inhibitory effect of the samples on NO production in lipopolysaccharide (LPS)‐stimulated RAW 264.7 macrophages was evaluated using the Griess reagent method [22]. Cells were seeded in 96‐well plates at a density of 0.5 × 10^5^ cells per well and incubated at 37°C in a humidified atmosphere containing 5% CO_2_ for 22 h. Following this incubation, samples at concentrations ranging from 3 to 25 μg/mL were added to the wells. After 30 min, 0.1 mg/mL of LPS (Sigma‐Aldrich, USA) was introduced to stimulate NO production, and the cells were further incubated for 24 h. After treatment, 100 μL of the culture supernatant was transferred to a new 96‐well plate and mixed with an equal volume of Griess reagent. The absorbance was measured at 570 nm using an iMark microplate reader (Bio‐Rad, USA). To assess cell viability, the remaining cells in the original plate were subjected to the MTT assay. This assay measures mitochondrial dehydrogenase activity in viable cells through the reduction of MTT (3‐(4,5‐dimethylthiazol‐2‐yl)‐2,5‐diphenyl tetrazolium bromide), providing an estimate of living cell numbers [23]. Cardamonin, a known inhibitor of NO production, served as the positive control.

### 2.9. Statistical Analysis

All RSM computations were conducted in Design‐Expert v12.0. Model adequacy was evaluated by analysis of variance (ANOVA) (model *F*, *p*), lack of fit, coefficients of determination (*R* ^2^, adjusted *R* ^2^, and predicted *R* ^2^), Adeq Precision, and coefficient of variation (CV). Data are reported as mean ± SD of triplicate runs.

## 3. Results and Discussion

### 3.1. The Process Range Conditions for the Extraction

To establish appropriate experimental ranges for a subsequent BBD optimization, a preliminary single‐factor study was conducted. This study aimed to investigate the influence of four key variables—ethanol concentration, temperature, solvent‐to‐material ratio, and extraction time—on the extraction efficiency of TPC and TSC from the fruit of *P. tectorius*.

The influence of EtOH concentration (%EtOH) is illustrated in Figure 1(a). The TPC yield from the fruit extract increased significantly with EtOH concentration, peaking at approximately 70% before plateauing. Similarly, the TSC yield rose with increasing EtOH content, reaching its maximum at 50% EtOH before declining at higher concentrations. This suggests that a hydroethanolic solvent is more effective than pure water or EtOH for extracting compounds from *P. tectorius* fruit, likely due to the varying polarities of the target phenolics and saponins. Based on these effects, the range of 0%–90% EtOH was selected as the operational range for the BBD.

Figure 1The effects of (a) ethanol concentration, (b) extracting temperature, (c) solvent‐to‐material ratio, and (d) extracting time on TPC and TSC of the extracts.

**Figure:**
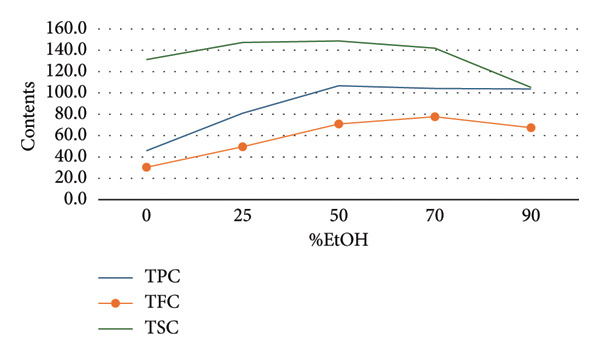
(a)

**Figure:**
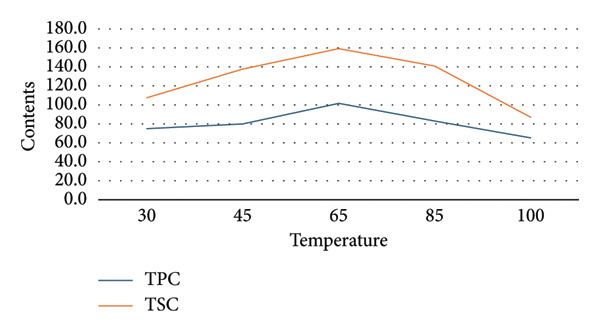
(b)

**Figure:**
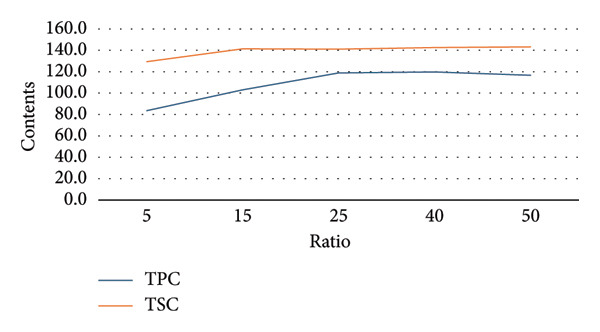
(c)

**Figure:**
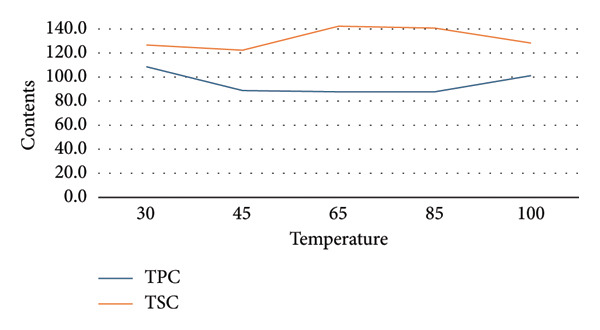
(d)

Regarding the extraction temperature, both TPC and TSC yields initially increased with temperature, reaching their optimal values at 65°C. At temperatures higher than this, a significant decrease in the yields of both compounds was observed. The data from the table show that at 85°C, the yields had already begun to decline from their peak. This decline is likely attributable to the degradation of thermosensitive substances within the fruit and the rapid evaporation of the EtOH. Therefore, to ensure compound stability and avoid degradation, the temperature range for the BBD was determined to be 30°C–80°C.

The solvent‐to‐material ratio demonstrated a strong positive correlation with extraction yield. A substantial increase in both TPC and TSC was observed when the ratio was increased from 5 to 25. A ratio below 10 was deemed unsuitable as the solvent volume was likely insufficient for proper wetting and extraction of the raw fruit material. Although yields continued to rise slightly as the ratio increased from 25 to 50, the rate of increase diminished, indicating marginal gains. To balance extraction efficiency with solvent consumption, the working range for the ratio in the BBD was established as 10–50 mL/g.

The extraction time showed different effects on TPC and TSC. The highest TPC was achieved at a shorter duration of 60 min, with longer times showing no improvement and potential degradation. Conversely, the TSC yield peaked at around 180 min. To accommodate the different optimal times for these two compound groups from *P. tectorius* while maintaining process efficiency, an extraction time range of 60–240 min was chosen as appropriate for the subsequent BBD experiments.

In conclusion, based on the single‐factor experimental results, the ranges for the four independent variables were selected for the BBD as follows: EtOH concentration (0%–90%), temperature (30°C–80°C), solvent‐to‐material ratio (10–50 mL/g), and time (60–240 min).

### 3.2. Optimize the Extraction Conditions Using BBD

The RSM with BBD was applied to determine the optimal condition for the extraction of phenolic compounds from *P. tectorius* fruits. The extraction design variable effects on the TPC and TSC values are given in Table 2.

**Table 2:** TPC and TSC of the extracts to independent variables using the Box–Behnken design.

No.	A: EtOH (%)	B: Temp. (°C)	C: Ratio (mL/g)	D: Time (min)	TPC (mgGAE/g)	TSC (mgAE/g)
1	45	30	30	60	58.3	132.8
2	45	30	50	150	74.5	140.2
3	90	30	30	150	91.5	111.2
4	45	30	30	240	73.1	140.5
5	45	55	10	60	65.2	138.5
6	90	55	30	60	116.6	125.9
7	90	80	30	150	112.3	142.2
8	45	30	10	150	56.8	121.4
9	90	55	50	150	121.5	136.7
10	0	55	30	240	47.3	135.2
11	45	80	30	240	94.5	164.2
12	90	55	50	60	116.2	124.4
13	45	80	30	60	105.6	152.3
14	90	55	30	150	111.3	129.9
15	0	55	50	150	50.2	131.1
16	0	55	30	60	47.9	128.9
17	45	55	30	150	90.3	161.5
18	45	80	50	150	111.2	170.6
19	90	55	30	240	119.3	128.7
20	45	80	10	150	87.8	149.8
21	0	30	30	150	27.8	110.8
22	45	55	30	150	92.8	155.2
23	45	55	50	240	100.7	160.1
24	0	80	30	150	58.7	142.6
25	45	55	30	150	99.3	162.8
26	45	55	10	240	70.5	139.3
27	45	55	30	150	87.6	162.4
28	90	55	10	150	96.3	121.6
29	45	55	30	150	96.4	158.9
30	0	55	10	150	35.5	123.5

### 3.3. The Optimal Model for TPC Enrichment

The experimental results from the BBD were analyzed using ANOVA to evaluate the effects of the process variables on the TPC yield. The statistical significance of the fitted quadratic model was checked by ANOVA. The experimental results from the BBD were analyzed using ANOVA to evaluate the effects of the process variables on the TPC yield. The ANOVA results indicate that the fitted quadratic model is highly significant, with a model F‐value of 55.56 and a *p* value < 0.0001, which implies that the model is highly suitable for describing the relationship between the independent variables and the TPC yield. Furthermore, the nonsignificant “lack of fit” (*p* = 0.4437) confirms that the model fits the experimental data well. The model’s suitability is also reinforced by its strong fit statistics. The coefficient of determination (*R* ^2^) of 0.9811 indicates that 98.11% of the variability in the response could be explained by the model, whereas the close agreement between the adjusted *R* ^2^ (0.9634) and predicted *R* ^2^ (0.9172) demonstrates good predictive power. Additionally, a high Adeq Precision of 27.1472 and a low CV % of 6.09% suggest an adequate signal‐to‐noise ratio and high experimental reliability. An analysis of the individual model terms revealed that the linear terms A (EtOH concentration), B (temperature), and C (ratio); the interaction term BD (temperature and time); and the quadratic terms A^2^, B^2^, and C^2^ all had a significant effect on TPC yield (*p* < 0.05). The significance of the quadratic terms confirms that the relationship is nonlinear. To improve and refine the model, it was proposed that nonsignificant interaction terms with *p* values greater than 0.5, specifically AD (*p* = 0.6711), BC (*p* = 0.5854), and CD (*p* = 0.8905), be eliminated to produce a more concise and robust model.

Following the initial analysis, the model was refined by removing the nonsignificant interaction terms (AD, BC, and CD) to produce a “reduced quadratic model” with markedly improved statistical significance and fit. The refined model’s *F*‐value increased substantially from 55.56 to 81.79, indicating it is now even more significant in explaining the relationship between the variables and the TPC yield, whereas the overall model *p* value remained highly significant (< 0.0001) and the lack of fit remained nonsignificant (*p* = 0.5396). The most notable improvements are seen in the fit statistics, which highlight the enhanced robustness and predictive power of this refined model. In particular, the adjusted *R* ^2^ increased from 0.9634 to 0.9684, and more importantly, the predicted *R* ^2^ increased from 0.9172 to 0.9430. This significantly narrowed the gap between the two values from 0.0462 to just 0.0254, indicating that removing the “noise” from irrelevant terms has made the model more accurate and reliable for making predictions. Furthermore, the Adeq Precision increased from 27.15 to 32.49, signifying an improved signal‐to‐noise ratio, and the CV % decreased from 6.09% to 5.66%, pointing to higher precision and reliability. Therefore, the model reduction process was highly successful, resulting in a more precise and robust model for the optimization of TPC extraction. The final regression equation to predict the TPC based on the actual values of the process variables is as follows:

(1) TPC=−86.00661.267132.450241.359430.261480.002240.0024160.002880.005580.011730.01480.00029+A+B+C+D−AB+AC−BD−A2−B2−C2−D2,

where A = EtOH (%), B = temperature (°C), C = ratio (mL/g), and D = time (min).

To visualize the relationship between the independent variables and the yield of TPC, three‐dimensional (3D) response surface plots were generated based on the model equation. These plots illustrate the interactive effects of two variables at a time on the TPC yield, whereas the other two variables are held constant at their central point. Figure 2(a) displays the interactive effect of EtOH concentration (A) and temperature (B) on TPC extraction. The plot reveals a significant curved surface, indicating that TPC yield increases with both variables up to an optimal region before leveling off. The peak TPC is predicted at high EtOH concentrations (approximately 70%–90%) and moderately high temperatures (approximately 70°C–80°C). This demonstrates a synergistic effect where higher temperatures enhance the solvent’s extraction capacity. For instance, at a constant temperature of 55°C, increasing the EtOH concentration from 0% to 90% (whereas other factors are held at their center points) raised the TPC yield from 47.9 mg GE/g to 111.3 mg GE/g. This trend is scientifically sound, as higher temperatures reduce solvent viscosity and increase the solubility and diffusion rate of phenolic compounds. At the same time, the appropriate EtOH concentration optimizes solvent polarity for extracting these target compounds. Figure 2(b) illustrates the relationship between EtOH concentration (A) and the solvent‐to‐material ratio (C). The response surface shows a clear positive correlation, where TPC yield consistently increases as both the EtOH concentration and the ratio are elevated. The steep incline suggests that both factors are strong drivers of extraction efficiency within the tested range. The experimental data support this, showing that at a fixed EtOH concentration of 45% and temperature of 30°C, increasing the ratio from 10 to 50 mL/g increased the TPC yield from 56.8 to 74.5 mg GE/g. This effect is primarily due to the principles of mass transfer; a larger volume of solvent (higher ratio) increases the concentration gradient between the solid material and the liquid phase, thereby promoting the diffusion of phenolic compounds into the solvent until equilibrium is approached.

Figure 2Response surfaces between (a) ethanol concentration and temperature, (b) ethanol concentration and solvent‐to‐material ratio, and (c) time and temperature to total phenolic contents of the *P. tectorius* fruit extracts.

**Figure:**
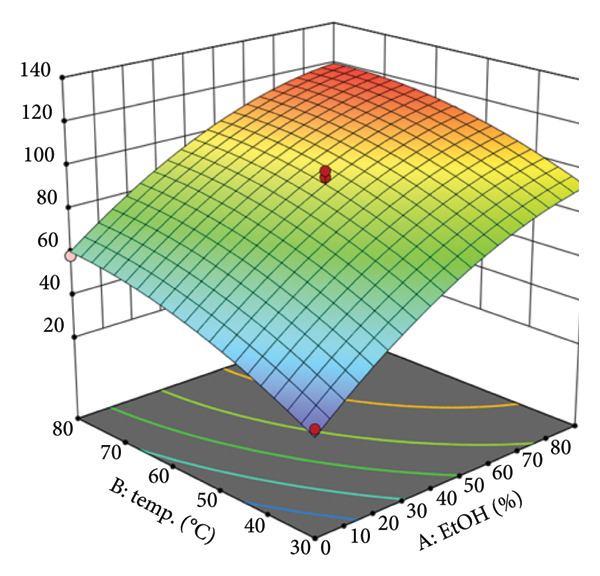
(a)

**Figure:**
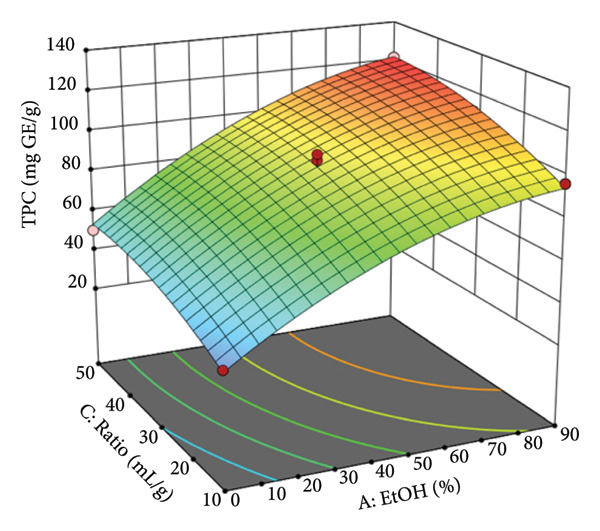
(b)

**Figure:**
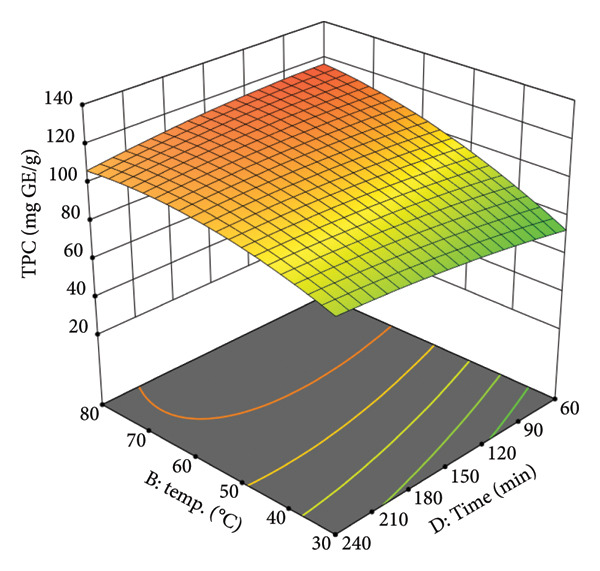
(c)

The interaction between temperature (B) and extraction time (D) is presented in Figure 2(c). The plot indicates that temperature is a more dominant factor than time in increasing TPC yield. However, a significant interaction between the two is evident. At lower temperatures (e.g., 30°C–40°C), extending the extraction time has a minimal effect on the TPC yield. In contrast, at higher temperatures (e.g., 80°C), a longer extraction time leads to a more substantial increase in TPC. This is supported by the data: At 80°C and 45% EtOH, the TPC yield was 111.2 mg GE/g after 150 min. The upward trend of the surface suggests that at elevated temperatures, which provide the necessary activation energy for extraction, a longer duration allows for more complete diffusion of solutes from the plant matrix.

### 3.4. The Optimal Model for TSC Enrichment

The results from the BBD for the TSC response were analyzed using ANOVA. The initial quadratic regression model was found to be highly significant, demonstrated by a model F‐value of 42.97 and a *p* value < 0.0001. Additionally, the “lack of fit” was not significant (*p* = 0.4082), indicating that there was no systematic error and the model was compatible with the experimental data. The high coefficient of determination (*R* ^2^ = 0.9757) also suggested that the model could explain 97.57% of the variability in the data. However, a more detailed analysis of the fit statistics revealed the necessity of refining the model to improve its predictive power. Specifically, a significant discrepancy was observed between the adjusted *R* ^2^ value of 0.953 and the predicted *R* ^2^ value of 0.8871. This difference of 0.0659 suggests that the model was likely overfitted, containing nonsignificant terms that contribute “noise.” An examination of the individual *p* values showed that several interaction terms, such as AB (*p* = 0.9123), AD (*p* = 0.6888), BC (*p* = 0.7832), and BD (*p* = 0.5652), had a very weak effect on the model. The presence of these terms reduces the model’s ability to accurately predict new outcomes.

Therefore, to enhance its reliability and predictive capability, the model was refined by eliminating the insignificant interaction terms with a *p* value greater than 0.5. This refinement process proved to be highly successful, resulting in a more robust and accurate model. The model *F*‐value increased sharply from 42.97 to 73.17, indicating a statistically stronger model. Most importantly, the difference between the adjusted *R* ^2^ (0.9614) and the predicted *R* ^2^ (0.932) was reduced significantly to just 0.0294. This confirms that the model was no longer overfitted and possessed excellent predictive capability. Furthermore, other statistical indicators also improved: The Adeq Precision increased from 21.68 to 27.82 and the CV % decreased to 2.31%, both of which confirm the enhanced strength and precision of the new model.

Based on the coefficients for the refined model, the final regression equation in terms of actual factors is

(2) TSC=30.659441.052811.848521.133880.1904490.0019580.0014460.012560.011810.017490.00065+A+B+C+D+AC+CD−A2−B2−C2−D2,

where A = EtOH (%), B = temperature (°C), C = ratio (mL/g), and D = time (min).

The response surface plots were constructed to visualize the interactive effects of the independent variables on the TSC. These plots are crucial for understanding the complex relationships and identifying the optimal conditions for extraction. Figure 3(a) illustrates the combined effect of EtOH concentration (A) and temperature (B). The surface plot is distinctly dome‐shaped, indicating that the TSC yield is maximized at intermediate levels of both variables. The yield increases as EtOH concentration rises from 0% and temperature increases from 30°C, reaching a peak before declining. This suggests that although a certain amount of EtOH and heat is beneficial for dissolving and extracting saponins, excessive levels can have an adverse effect. This may be due to changes in solvent properties, such as polarity, at very high EtOH concentrations or potential solvent loss at temperatures approaching the boiling point of EtOH, which would alter the extraction conditions. The experimental data confirm this, showing that at a fixed ratio and time, the TSC yield at 45% EtOH and 80°C (170.6 mg AE/g) is significantly higher than at 90% EtOH and 80°C (152.3 mg AE/g), highlighting the existence of an optimal range. The interaction between EtOH concentration (A) and solvent‐to‐material ratio (C) is depicted in Figure 3(b). Similar to the previous plot, this response surface also shows a clear optimal region. The TSC yield increases as both the EtOH concentration and the ratio are increased, but only up to a certain point. The curvature indicates that after reaching an optimal EtOH concentration (around 40%–50%) and ratio (around 30–40 mL/g), the TSC yield begins to plateau or even slightly decrease. This demonstrates the significant interactive effect between these two variables. For example, at a fixed temperature of 55°C, increasing the ratio from 10 to 50 mL/g at 45% EtOH shows a significant increase in yield (from 158.9 to 162.4 mg AE/g), underscoring the importance of an adequate solvent volume to facilitate mass transfer.

Figure 3Response surfaces between (a) ethanol concentration and temperature, (b) ethanol concentration and solvent‐to‐material ratio, and (c) time and solvent‐to‐material ratio to total saponin contents of the *P. tectorius* fruit extracts.

**Figure:**
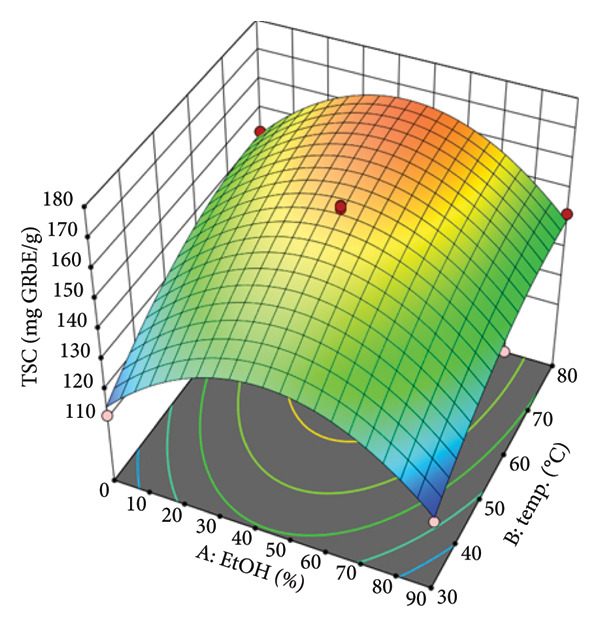
(a)

**Figure:**
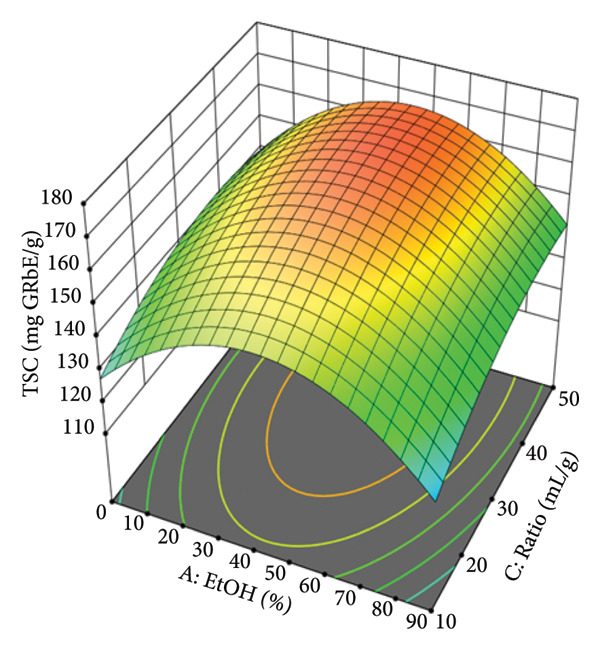
(b)

**Figure:**
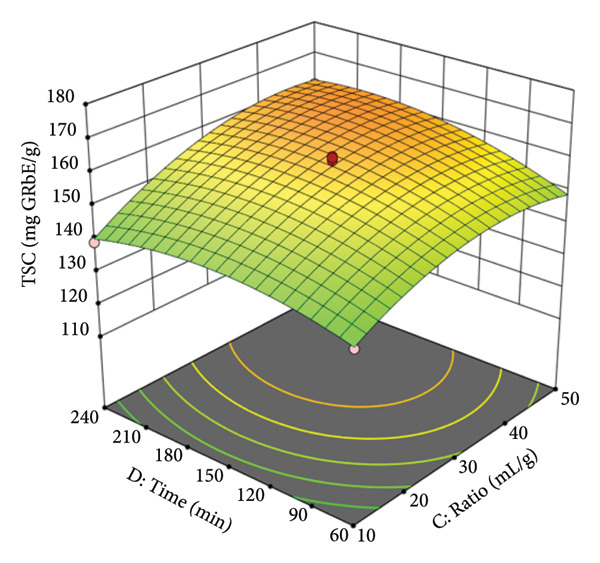
(c)

Figure 3(c) presents the interactive effect of extraction time (D) and solvent‐to‐material ratio (C). The surface shows that both factors have a positive effect on the TSC yield, with the yield increasing as both time and ratio are extended. The slope is steeper for the ratio than for time, suggesting the solvent volume is a more dominant factor in this interaction. The plot shows a continuous rise toward the upper limits of both variables, with the highest TSC yields found at longer times (around 180–240 min) and higher ratios (around 40–50 mL/g). This is consistent with mass transfer principles, where a larger solvent volume and a longer contact time allow for more complete diffusion of the saponins from the plant matrix into the solvent, leading to a higher extraction yield. For instance, at 45% EtOH and 55°C, increasing the extraction time from 60 min to 240 min at a ratio of 50 mL/g resulted in an increased yield from 150.2 mg/g to 160.1 mg AE/g.

The extraction time exerted contrasting effects on phenolic and saponin recovery due to their distinct physicochemical properties. Phenolic compounds, being relatively small and highly soluble in aqueous ethanolic media, reach diffusion equilibrium rapidly, and prolonged heating may promote oxidative degradation or polymerization. In contrast, saponins are amphiphilic glycosides with bulky triterpenoid or steroidal aglycones, which require extended contact time for complete solvent penetration and micellar solubilization. Consequently, phenolic yield tends to plateau or slightly decline with time, whereas saponin yield continues to increase until reaching its own saturation point.

### 3.5. Optimize the Extraction Conditions

The refined regression models for both TPC and TSC were utilized to determine the optimal conditions for extracting phytochemicals from *P. tectorius* fruit. As the ideal conditions for maximizing TPC and TSC differ, a multiresponse optimization was performed using the numerical optimization feature of the software Design‐Expert. This approach allows for finding a range of ideal conditions by assigning different “importance levels” to each response, providing flexibility based on the desired outcome.

Table 3 summarizes seven potential optimal solutions generated by varying the importance placed on TPC versus TSC. The results show a clear trade‐off between the two responses. Conditions prioritizing TPC, designated as the “Opt_TPC” series, consistently require a high EtOH concentration between 70% and 90% and a high temperature of approximately 80°C. Conversely, conditions prioritizing TSC, the “Opt_TSC” series, favor a lower EtOH concentration around 45%–55% and significantly longer extraction times exceeding 175 min, whereas the optimal temperature remains high. The “balance” condition represents a compromise, using intermediate parameters to achieve good, though not maximal, yields of both TPC and TSC simultaneously. This analysis provides a set of validated optimal conditions, allowing for the selection of specific extraction parameters depending on whether the desired final product is an extract rich in phenolics, saponins, or a balanced combination of both.

**Table 3:** Summary of predicted optimal conditions for achieving different extraction goals, prioritizing either TPC, TSC, or a balance of both responses.

Condition	Importance level		%EtOH (%)	Temp. (°C)	Ratio (mL/g)	Time (min)
	TPC	TSC				
Opt_TPC1	5	0	90.0	80.0	48.2	104.0
Opt_TPC2	4	1	76.6	80.0	48.6	128.1
Opt_TPC3	3	2	68.9	79.1	47.2	146.4
Balance	2.5	2.5	59.3	78.3	45.5	167.0
Opt_TSC 1	2	3	55.0	78.1	44.7	175.1
Opt_TSC 2	1	4	49.1	78.1	43.6	186.0
Opt_TSC 3	0	5	45.3	78.3	42.9	193.1

To confirm the validity and predictive accuracy of the developed models, a series of validation experiments was conducted. The seven sets of optimal conditions derived from the numerical optimization were slightly adjusted for practical convenience in a laboratory setting. Extractions were then performed in triplicate under these adjusted conditions. Table 4 presents a comparison between the TPC and TSC yields predicted by the regression models and the values obtained through these validation experiments.

**Table 4:** Predicted versus experimental yields (mean ± SD, *n* = 3) of TPC (mg GAE/g) and TSC (mg AE/g) for the validation of the seven adjusted optimal extraction conditions.

Samples	EtOH (%)	Temp. (°C)	Ratio (mL/g)	Time (min)	Predict values		Experimental	
					TPC	TSC	TPC	TSC
Opt_TPC1	90	80	48	104	129.3	138.5	124.8 ± 8.8	126.1 ± 12.7
Opt_TPC2	75	80	48	130	124.2	155.2	121.1 ± 11.1	142.8 ± 16.5
Opt_TPC3	65	80	47	150	118.8	163.0	118.3 ± 6.5	151.8 ± 15.9
Balance	60	80	46	170	115.0	166.3	114.7 ± 7.1	162.4 ± 19.6
Opt_TSC1	55	80	45	175	111.7	168.0	109.7 ± 8.8	168.3 ± 15.2
Opt_TSC2	50	80	44	190	107.4	169.2	106.2 ± 11.8	178.9 ± 12.1
Opt_TSC3	45	80	43	195	103.5	169.5	105.6 ± 8.5	185.6 ± 10.6

The results demonstrate a strong correlation and excellent agreement between the predicted and experimental values across all seven tested conditions, affirming the reliability of the optimization process. In particular, the experimental data closely matched the values predicted by the models. For instance, the “balance” condition showed remarkable accuracy, with an experimental TPC of 114.7 ± 7.1 mg GAE/g against a prediction of 115.0 mg GAE/g. Furthermore, the analysis confirmed that the Opt_TSC3 condition, which was designed to maximize saponin content, successfully yielded the highest experimental TSC of 185.6 ± 10.6 mg AE/g and concurrently the lowest experimental TPC of 105.6 ± 8.5 mg GAE/g. This lowest TPC value showed excellent agreement with its predicted value of 103.5 mg GAE/g. Although the highest experimental TSC was greater than its predicted value of 169.5 mg AE/g, the model nonetheless accurately identified the specific conditions required to achieve the maximum saponin yield.

The experimental data also validated the predicted trade‐off between the two responses; conditions designed to favor TPC resulted in extracts with higher experimental TPC yields, whereas conditions prioritizing TSC successfully produced extracts richer in saponins. The close correspondence between the predicted and actual results validates the accuracy of the regression models. This confirms that the developed models are reliable and effective tools for navigating the design space and optimizing the extraction of both phenolic and saponin compounds from *P. tectorius* fruit.

### 3.6. Bioactivities of the Extracts

#### 3.6.1. Antioxidant and NO Production Inhibitory Effect

The antioxidant activity of the seven extracts was evaluated through their ability to scavenge DPPH and hydroxyl radicals, with the results presented as IC_50_ values. The data revealed a clear correlation between antioxidant activity and the TPC of the extracts. The samples belonging to the “Opt_TPC” series, which were optimized for high TPC, were the only ones to exhibit significant activity with IC_50_ values below 100 μg/mL in both assays. Among them, the “Opt_TPC1” extract demonstrated the highest potency, with an IC_50_ value of 76.4 μg/mL for DPPH scavenging and 62.5 μg/mL for hydroxyl radical scavenging. In contrast, the saponin‐optimized extracts (“Opt_TSC” series) and the “balance” extract showed considerably weaker activity, with IC_50_ values exceeding 100 μg/mL in most tests. Although the TPC‐rich extracts demonstrated notable antioxidant potential, their activity was lower than that of the pure compound positive controls. Specifically, the DPPH scavenging activity of the most potent extract (“Opt_TPC1”) was less than that of ascorbic acid (IC_50_ = 27.4 μg/mL), and its hydroxyl radical scavenging activity was less than that of catechin (IC_50_ = 31.7 μg/mL). This result is expected, as crude extracts are complex mixtures, whereas the controls are highly active, pure antioxidant compounds. These findings collectively suggest that the phenolic constituents are the primary contributors to the antioxidant capacity of the *P. tectorius* extracts.

The anti‐inflammatory potential of the seven optimized *P. tectorius* fruit extracts was evaluated by measuring their ability to inhibit NO production in LPS‐stimulated cells (Table 5). A clear trend emerged regarding the extracts’ potency, which was found to correlate with the saponin contents.

**Table 5:** Free‐radical scavenging and NO production inhibitory effects of *P. tectorius* fruit extracts under optimal conditions.

Samples	DPPH (IC_50_, μg/mL)	Hydroxyl (IC_50_, μg/mL)	NO inhibition (IC_50_, μg/mL)
Opt_TPC1	76.4 ± 3.8^a^	62.5 ± 4.1^d^	91.3 ± 8.2^i^
Opt_TPC2	82.1 ± 4.5^b^	70.8 ± 5.3^e^	85.4 ± 6.6^j^
Opt_TPC3	89.5 ± 5.1^b^	79.1 ± 6.2^f^	84.2 ± 7.3^j^
Balance	> 100	95.6 ± 8.1^g^	75.5 ± 3.9^k^
Opt_TSC 1	> 100	> 100	68.8 ± 1.1^l^
Opt_TSC 2	> 100	> 100	ND
Opt_TSC 3	> 100	> 100	ND
Ascorbic acid^∗^	27.4 ± 1.6^c^	—	—
Catechin^∗∗^	—	31.7 ± 2.8^h^	—
Cardamonin^#^			3.1 ± 0.4^m^

^∗,∗∗,#^Positive control.

^a–m^Data are expressed as mean ± SD. Means in each column with different letters are significantly different (*p* < 0.05).

The IC_50_ values ranged from 91.3 μg/mL for the TPC‐rich extract Opt_TPC1 down to 68.8 μg/mL for the saponin‐rich extract Opt_TSC1, indicating that Opt_TSC1 was the most potent anti‐inflammatory agent among the tested samples. As expected, all crude extracts were less potent than the pure compound positive control, cardamonin, which had an IC_50_ of 3.1 μg/mL. To ensure that the observed NO inhibition was not a result of cytotoxicity, cell survival was assessed (Table S1). The extracts from the Opt_TPC series, the balance condition, and Opt_TSC1 all demonstrated excellent safety profiles, with cell survival rates consistently above 92%. In contrast, the Opt_TSC2 and Opt_TSC3 extracts, which contained the highest concentrations of saponins, exhibited significant cytotoxicity, causing cell survival to drop to as low as 64.3%. Consequently, the IC_50_ values for these two extracts could not be determined. This finding suggests that although saponin‐rich extracts possess greater anti‐inflammatory potency, very high concentrations of saponins or coextracted compounds may induce a cytotoxic effect on the RAW 264.7 cells. Phenolic compounds exert antioxidant effects mainly through electron or hydrogen donation and stabilization of resulting phenoxyl radicals, as well as through transition‐metal chelation (Fe^2+^/Cu^2+^) that limits Fenton‐type radical formation [24, 25]. In contrast, saponins are amphiphilic glycosides whose aglycone cores and membrane‐active properties modulate inflammatory signaling and suppress LPS‐induced NO production via iNOS/NF‐κB downregulation, while stabilizing cellular membranes [26]. These class‐specific properties explain why TPC‐enriched extracts excel in antioxidant assays, whereas TSC‐enriched extracts more effectively inhibit NO production. Among the optimized conditions, Opt_TPC1 and Opt_TSC1 displayed the highest bioactivities within their groups. The 90% EtOH and moderate extraction time (104 min) in Opt_TPC1 favored recovery of midpolarity flavonoids and aldehydes with strong redox capacity, accounting for its lower IC_50_ values despite similar TPC yields to Opt_TPC2–3. Conversely, Opt_TSC1 (55% EtOH, 175 min) likely promoted the extraction of moderately polar triterpenoid saponins with stronger membrane‐modulating activity, whereas higher water content in Opt_TSC2–3 may have diluted or degraded active aglycones. Overall, the superior potency of these extracts reflects not only quantitative yield but also qualitative compositional shifts controlled by solvent polarity and extraction kinetics, underscoring the need to balance concentration and selectivity in RSM optimization.

The optimized phenolic‐ and saponin‐enriched extracts of *P. tectorius* thus exhibit distinct functional profiles with promising industrial relevance. Phenolic‐rich extracts could serve as natural antioxidants in food and nutraceutical products, whereas saponin‐rich extracts, showing anti‐inflammatory and mild surfactant properties, may be applicable in cosmetic or pharmaceutical formulations. Further work on scale‐up, stability, and formulation compatibility would support their practical use.

## 4. Conclusion

This study successfully optimized the extraction conditions for phenolic‐ and saponin‐rich fractions from *P. tectorius* fruits using RSM based on a BBD. The developed quadratic models exhibited excellent statistical reliability, with coefficients of determination (*R* ^2^) exceeding 0.98 for both TPC and TSC responses, confirming the adequacy of the fitted models. Under the validated optimal conditions, the experimental yields reached 124.8 ± 8.8 mg GAE/g for total phenolics and 168.3 ± 15.2 mg AE/g for total saponins, closely matching the predicted values. The optimized phenolic extract (Opt_TPC1) demonstrated potent antioxidant effects with DPPH and hydroxyl radical IC_50_ values of 76.4 and 62.5 μg/mL, respectively, whereas the saponin‐rich extract (Opt_TSC1) showed considerable anti‐inflammatory activity with an IC_50_ of 68.8 μg/mL for NO inhibition. These results highlight the compositional and functional complementarity between phenolic and saponin fractions and support the potential of *P. tectorius* fruit as a renewable source of bioactive ingredients. Nevertheless, the cytotoxicity observed in extracts with excessive saponin content indicates a need for further refinement. Future studies should focus on isolating individual active compounds, improving extract selectivity, and conducting comprehensive safety assessments to ensure both efficacy and biocompatibility in prospective food, cosmetic, and pharmaceutical applications.

## Conflicts of Interest

The authors declare no conflicts of interest.

## Funding

This study was funded by the Vietnam Academy of Science and Technology (VAST) under the Grant Number NCXS 01.02/23‐25.

## Supporting Information

Table S1. NO inhibition screening data.

## Supporting information


**Supporting Information** Additional supporting information can be found online in the Supporting Information section.

## Data Availability

The data supporting the findings of this study are included in this published article and its supporting information files.
