# Supplementary material for: Optimization of Phenolic‐ and Saponin‐Enriched Extraction From Pandanus tectorius Fruit Using Box–Behnken Design and Evaluation of Their Bioactivities
Source: J Anal Methods Chem. 2025 Dec 9;2025:5539843. doi: 10.1155/jamc/5539843 (PMC12767014; doi:10.1155/jamc/5539843)
Supplement: Supplementary file 1 — Supporting Information Additional supporting information can be found online in the Supporting Information section. [file JAMC-2025-5539843-s001.docx]

**Supplemental**

**Optimization of Phenolic- and Saponin-Enriched Extraction from *Pandanus tectorius* Fruit Using Box–Behnken Design and Evaluation of Their Bioactivities**

Do Hoang Giang^1,2^, Nguyen Hai Dang^1^, Tran Thi Thu Phuong^1^, Le Thanh Huong^1^, Nguyen Thu Uyen^2^, Nguyen Thi Luyen^2^, Nguyen Thi Thu Thuy^3^, Hoang Le Tuan Anh^2^, Nguyen Ngoc Tung^2^, Nguyen Tien Dat^2,*^

^1^ University of Science and Technology of Hanoi, Vietnam Academy of Science and Technology (VAST), 18-Hoang Quoc Viet, Cau Giay, Hanoi 10000, Vietnam

^2^ Center for High Technology Research and Development, VAST, 18-Hoang Quoc Viet, Cau Giay, Hanoi 10000, Vietnam.

^3^ Joint Vietnam-Russia Tropical Science and Technology Research Center, Nguyen Van Huyen, Cau Giay, Hanoi 10000, Vietnam

* Correspondence should be addressed to Nguyen Tien Dat, email: ngtiend@gmail.com

# Table S1. NO inhibition screening data

| **Samples** | **C (µg/mL)** | **%I** | **%Cell Survival** | **IC50 (µg/mL)** |
| --- | --- | --- | --- | --- |
| Opt_TPC1 | 25 | 16.1 ± 5.3 | 99.8 ± 1.5 | 91.3 ± 8.2 |
|  | 100 | 53.7 ± 1.5 | 97.9 ± 1.4 |  |
| Opt_TPC2 | 25 | 28.2 ± 0.7 | 101.3 ± 4.7 | 85.4 ± 6.6 |
|  | 100 | 57.5 ± 6.7 | 99.8 ± 3.5 |  |
| Opt_TPC3 | 25 | 15.9 ± 8.9 | 99.2 ± 0.2 | 84.2 ± 7.3 |
|  | 100 | 57.8 ± 3.9 | 94.6 ± 5.3 |  |
| Balance | 25 | 31.6 ± 4.6 | 96.1 ± 3.0 | 75.5 ± 3.9 |
|  | 100 | 58.1 ± 3.9 | 94.3 ± 4.8 |  |
| Opt_TSC 1 | 25 | 36.1 ± 2.5 | 97.6 ± 5.3 | 68.8 ± 1.1 |
|  | 100 | 64.8 ± 3.6 | 92.8 ± 2.3 |  |
| Opt_TSC 2 | 25 | 64.8 ± 3.6 | 85.8 ± 7.3 | ND |
|  | 100 | 48.4 ± 2.6 | 68.5 ± 4.3 |  |
| Opt_TSC 3 | 25 | 42.9 ± 0.9 | 64.3 ± 1.3 | ND |
|  | 100 | 42.9 ± 7.9 | 64.4 ± 0.6 |  |
| Cardamonin^#^ | 1.25 | 41.3 ± 4.5 | 100.0 ± 4.1 | 3.1 ± 0.4 |
|  | 5 | 83.9 ± 6.7 | 97.8 ± 3.8 |  |
| ^#^Positive control, ND: not detected due to high toxicity to the cells | | | | |
